# Intermediate Pond Sizes Contain the Highest Density, Richness, and Diversity of Pond-Breeding Amphibians

**DOI:** 10.1371/journal.pone.0123055

**Published:** 2015-04-23

**Authors:** Raymond D. Semlitsch, William E. Peterman, Thomas L. Anderson, Dana L. Drake, Brittany H. Ousterhout

**Affiliations:** University of Missouri, Division of Biological Sciences, Columbia, Missouri, United States of America; Institute of Agronomy, University of Lisbon, PORTUGAL

## Abstract

We present data on amphibian density, species richness, and diversity from a 7140-ha area consisting of 200 ponds in the Midwestern U.S. that represents most of the possible lentic aquatic breeding habitats common in this region. Our study includes all possible breeding sites with natural and anthropogenic disturbance processes that can be missing from studies where sampling intensity is low, sample area is small, or partial disturbance gradients are sampled. We tested whether pond area was a significant predictor of density, species richness, and diversity of amphibians and if values peaked at intermediate pond areas. We found that in all cases a quadratic model fit our data significantly better than a linear model. Because small ponds have a high probability of pond drying and large ponds have a high probability of fish colonization and accumulation of invertebrate predators, drying and predation may be two mechanisms driving the peak of density and diversity towards intermediate values of pond size. We also found that not all intermediate sized ponds produced many larvae; in fact, some had low amphibian density, richness, and diversity. Further analyses of the subset of ponds represented in the peak of the area distribution showed that fish, hydroperiod, invertebrate density, and canopy are additional factors that drive density, richness and diversity of ponds up or down, when extremely small or large ponds are eliminated. Our results indicate that fishless ponds at intermediate sizes are more diverse, produce more larvae, and have greater potential to recruit juveniles into adult populations of most species sampled. Further, hylid and chorus frogs are found predictably more often in ephemeral ponds whereas bullfrogs, green frogs, and cricket frogs are found most often in permanent ponds with fish. Our data increase understanding of what factors structure and maintain amphibian diversity across large landscapes.

## Introduction

One of the most fundamental questions in ecology is what maintains species diversity [1]. Most ecologists agree that spatial and temporal variation in environmental conditions play major roles in maintaining diversity. Historically, gradients of environmental disturbance and productivity have been influential in explaining patterns of diversity [2–7]. However, a number of different patterns have been observed between diversity, disturbance, and productivity based on empirical studies [7,8], and the lack of consistency has raised questions about the usefulness of single over-arching hypotheses predicting diversity in natural communities [7,9]. More important than focusing on a single pattern or hypothesis, however, is a detailed understanding of the actual mechanisms shaping diversity patterns in nature [7,10]. Further, understanding that multiple gradients of disturbance and productivity likely all interact with species traits to generate patterns of diversity we see in the real world is also critical [7,11]. This is especially important because natural or anthropogenic environmental disturbances such as wildfire, flooding, grazing, invasive species or droughts due to climate change can alter the frequency, intensity, duration, and timing of disturbance regimes making a mechanistic understanding critical for future management and conservation of species communities.

Communities of pond-breeding amphibians reproduce in a range of aquatic habitats varying from highly ephemeral–desert pools, small road ruts, ditches, and borrow-pits–to seasonal–wetlands and marshes–to more permanent and stable–bogs, farm ponds, and glacial lakes [12–14]. Much of amphibian life history, including breeding migrations, phenology, oviposition, hatching, larval period, and morphological fitness traits, is driven by disturbance regimes and may influence community structure [13,14]. Disturbance along a gradient of pond drying (or hydroperiod = length of time a pond holds water) has been shown to be important in explaining both species presence and reproductive success [15–17]. A synthesis of community structure across freshwater gradients such as those used by amphibians revealed that hydroperiod (i.e., physical factor) interacts with species traits (i.e. biotic factor) to predict species success [18]. Further, this synthesis provided an understanding of how species traits interact with disturbance to determine their survival thereby leading to predictable community structure [18]. Thus, examining a range of pond environments with different probabilities of drying across a large landscape inhabited by a diversity of amphibian species can yield insight into the mechanisms driving species success, predict the structure of communities, and help develop management and protection guidelines for maintaining biodiversity.

Here, we present data on amphibian abundance and diversity collected across a large landscape of 7140 ha consisting of 200 ponds that represent most of the possible lentic aquatic breeding habitats common in this region and encompass a wide-range of sizes and disturbance processes. Our data also represent the disturbance frequency and intensity commonly experienced by pond-breeding amphibians throughout Missouri [19], and likely the Midwestern region of the U.S. in general. Therefore, we believe our study includes a wide range of ecological processes that can be missing from studies where sampling intensity is low, rare species are not detected, sample area is small, or partial disturbance gradients are sampled [8]. Our first objective was to test the coarse-scale relationship between pond area and density, species richness, and diversity of amphibian with predictions that pond drying and predation are important ecological drivers [18]. Because ponds are breeding resources for amphibians, longer hydroperiods due to pond area, increased depth or volume can increase success of larvae by decreasing disturbance of drying and result in greater abundance and diversity. However, as pond hydroperiod increases, the probability of fish invasion and invertebrate predator density also increases, and a second type of disturbance from large predators [2,5] such as fish increases consumption, lowering abundance and diversity. Thus, we hypothesize that a balance between pond drying and predation is important for maximizing abundance and diversity. Secondly, if the predicted quadratic relationship between pond area and abundance, diversity, and richness is revealed, we present more detailed fine-scale analyses of the intermediate size ponds to discover ecological drivers of abundance and diversity. Our ultimate goal is to provide an analysis of a large-scale pattern based on extensive field surveys and provide general mechanistic inferences regarding the balance between hydroperiod and predation pressure that can be used to develop management and conservation guidelines.

## Materials and Methods

### Ethics Statement

This research was conducted in compliance with all laws and regulations for the state of Missouri and the USA, and was conducted under Missouri Wildlife Collector’s permits 15602, 15562, and sampling was approved by the University of Missouri Animal Care and Use Committee Protocol 7403.

### Data availability

The data used in analyses for this manuscript are available at Figshare in separate files for abundance and richness—diversity (http://dx.doi.org/10.6084/m9.figshare.1320840).

### Study Area

**Fig 1:**
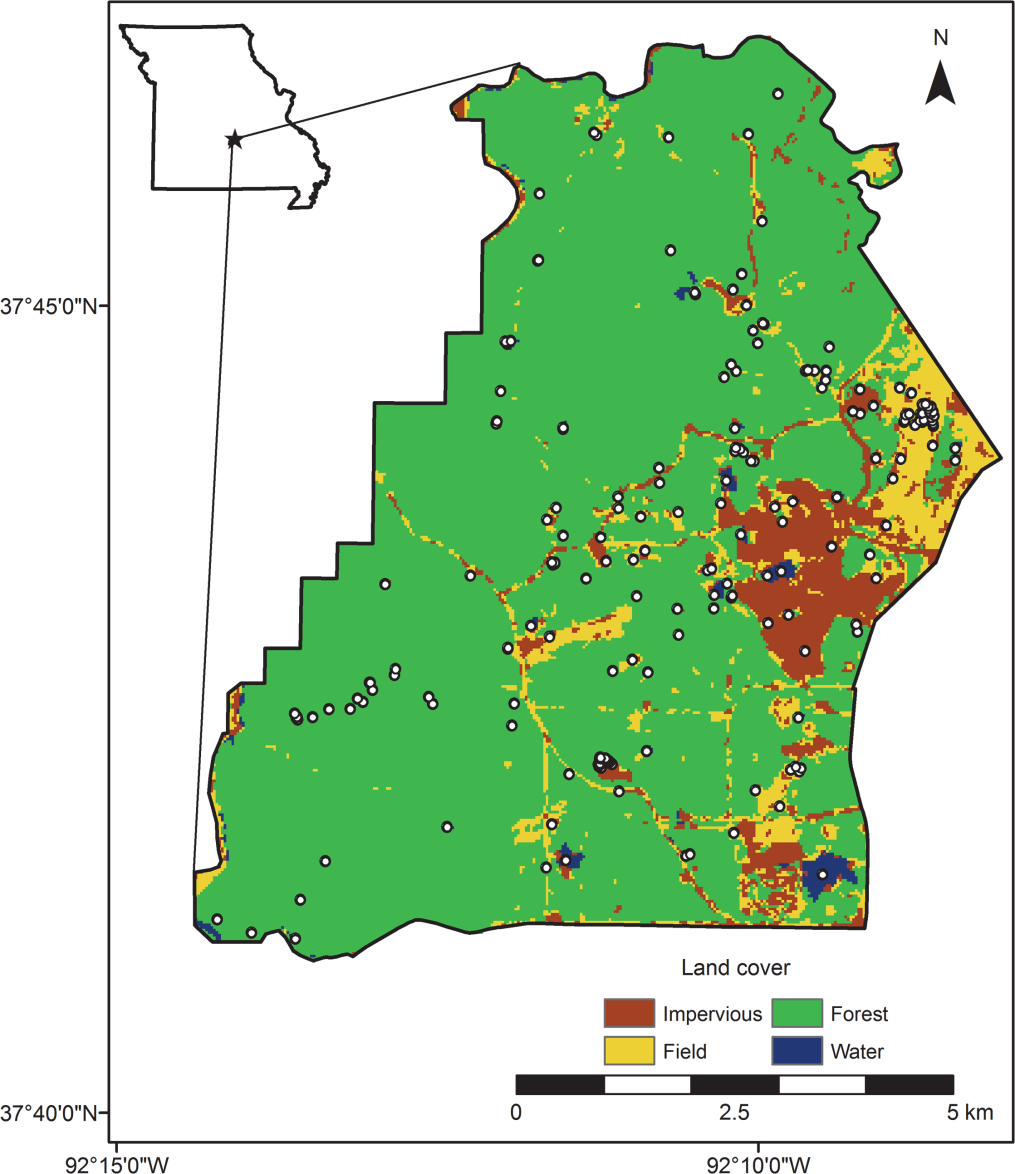
Map of the sampled landscape (7,140 ha) within Fort Leonard Wood (FLW), Missouri. Major land cover types are indicated by different colors and all known ponds (white dots) are shown.

**Fig 2:**
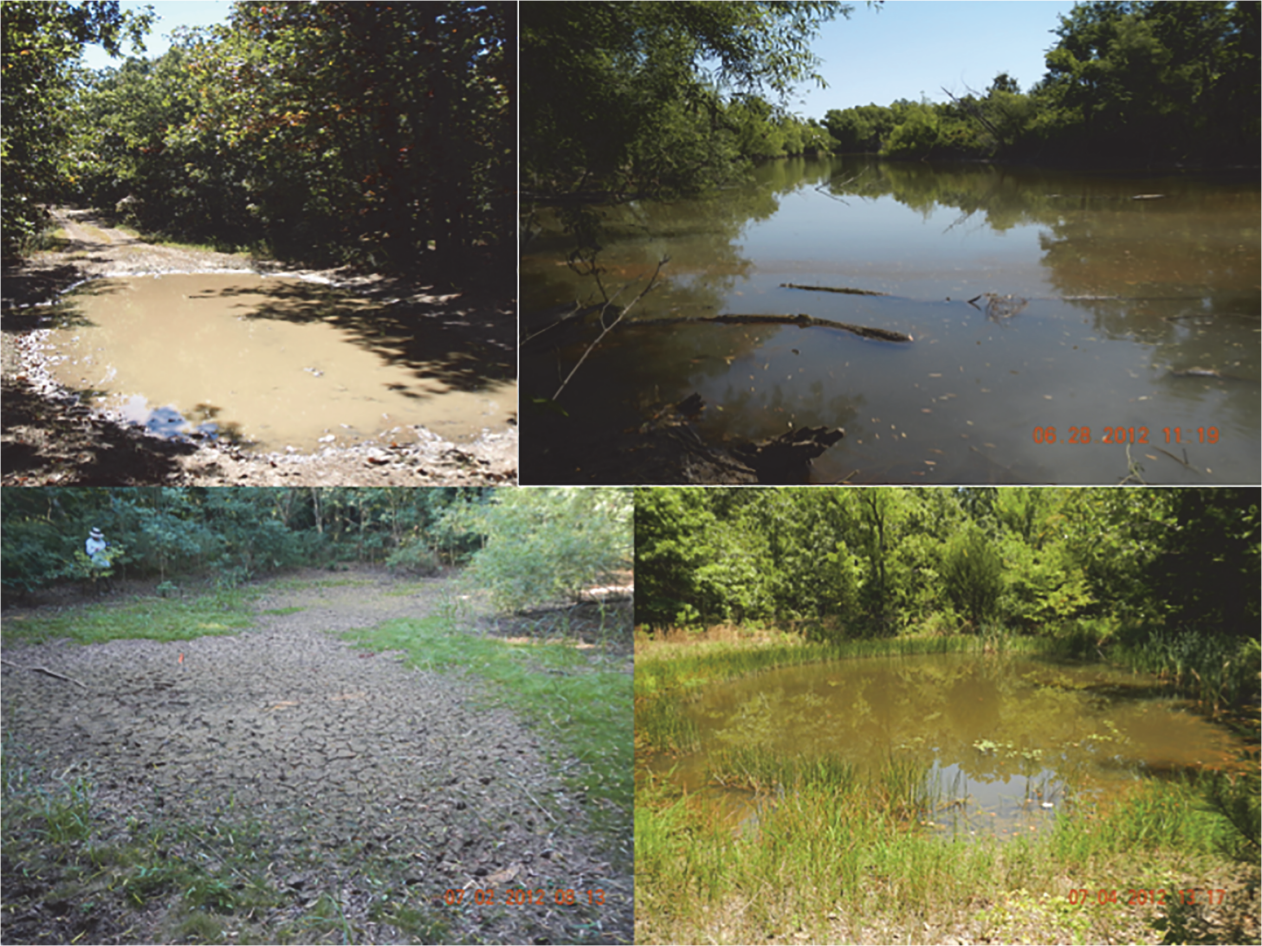
Examples of water bodies sampled for larval amphibians at Fort Leonard Wood (FLW), Missouri. Road rut (upper left), permanent farm pond (upper right), seasonal pond (lower right), and a dried seasonal pond (lower left).

We sampled amphibian breeding ponds at Fort Leonard Wood (FLW), an active U.S. military training base in the Ozark Highlands of Pulaski County, Missouri (37.92°N, 92.17°W; Fig 1) [20]. FLW encompasses 24,686 hectares that is 80% forested, and has an extensive road system throughout much of the military base. The site contains >500 constructed wildlife ponds, farm ponds, and unintentional water bodies (i.e. sedimentation basins, tire ruts, roadside ditches, and impact craters), hereafter “ponds” (Fig 2). Many of the ponds were fishless, constructed wildlife ponds that were excavated using construction equipment, often < 0.04 hectares in area, range from 1–80 years in age, and have a constructed berm to retain water. Wildlife ponds were originally constructed for other wildlife (e.g., turkey and deer) as a source of water but have been naturally colonized by up to 16 species of amphibians in Missouri [19,21]. Some large (>1 hectare) ponds and small lakes are stocked with game fish, mosquitofish, or have been naturally colonized from nearby perennial streams. Tire rut ponds at FLW are water bodies formed on unpaved roads as a result of vehicle traffic, current or historic. In this study, we intensively sampled a subset of all ponds (n = 200) in a representative 7,140-hectare area of the west-central portion of the base (Figs 1 and 2) [20]. We searched the area to include all possible amphibian breeding sites. The sampled ponds and surrounding habitat are representative of FLW and the broader region, and include contiguous forest, open fields, and human activities (e.g., vehicle traffic and building construction; Fig 2). Because of the extensive loss of natural wetlands in Missouri and many regions of the U.S. [22], constructed ponds are the dominant breeding resource for nearly all species in Missouri [19,23], and in parts of other states such as Kentucky, Tennessee, Iowa, and Illinois (R. Semlitsch, pers. obs.).

### Biotic Sampling

Ponds were sampled from September through July each year (2012, 2013) as part of other studies on the egg, larval, and metamorph life stages of several target species [20,24]. In this study, we focused our sampling on the aquatic larval stage that represented successful reproduction of a species at a pond. Each pond (n = 200) was intensively sampled during two survey periods to encompass the larval period of all known species, once over five weeks in February-March and again during three weeks in May/June in both 2012 and 2013. During each visit, each pond holding water was surveyed on three successive days using two methods (dip-net sweeps and funnel traps) [20,23] to maximize detection of rare species. Thus, during the three days, six samples were obtained (3 days of funnel trap collections plus 3 days of dip-net collections). Only one pond-breeding amphibian species known at FLW, but rare (*Gastrophryne carolinensis*) was missed in our surveys. Funnel traps (3mm mesh size, 38 x 26 x 26 cm; Memphis Net and Twine) were deployed at each site when water was deep enough to cover trap openings and when site access could be guaranteed for the survey period. The number of traps was scaled to pond surface area (one per 25 m^2^ of pond surface area [23]), with a maximum of 20 traps per pond. Traps were checked daily (maximum of 60 trap checks over three days) to count the number of amphibian larvae (identified to species) and other aquatic animals (see below), after which all animals were returned to the point of capture. The number of dip-net sweeps per pond (40 x 35 cm dip-net, 3.2 mm mesh) was scaled identically to funnel traps with up to 20 sweeps per day, 60 maximum over three days. Dip-net sweeps were approximately 1.5m in length, and included leaf litter and vegetation in samples [25]. Trapping and dip-netting occurred within 2 m of the shoreline, and in all aquatic habitat types present (e.g., cattails, emergent grass) to minimize bias. Some ponds were precluded from funnel trapping due to shallow water depth or other logistical constraints and only dip-net sweeps were performed at those sites (n = 19). Fish presence/absence was recorded as they were captured in traps and dip nets; fish were never observed at a pond when they were not also captured during sampling. However, our sampling techniques did not allow for fish diversity and biomass estimates. Invertebrate predators, separated into functional groups (often family), were counted and released similarly to amphibians (S1 Table).

### Habitat sampling

Pond attributes were also measured either at the time of sampling each year or for static variables once during the summer of 2012 (see summary of pond habitat parameters assessed in Table 1 from Ref 20). Pond diameter was measured across the longest and shortest axes using an infrared range finder (Bushnell Yardage Pro) and multiplied together to estimate area. Because of the varied origin of ponds at FLW, the water bodies we sampled varied greatly in shape and area was best characterized by a rectangle. Hydroperiod was assigned into four categories on a continuum: ephemeral (dries multiple times every year), summer (dries once a year during the summer), semi-permanent (dries in drought years), and permanent (never dries). These categories were assigned based on multiple visits to all ponds that occurred approximately every other month during 2012 and 2013 during other studies, deployment of temperature loggers to detect drying date, and from historical data of the ponds (K. Lohraff, FLW Wildlife Manager, pers. comm.). Hydroperiod was treated as a continuous variable in all analyses. Canopy closure was estimated using a spherical densitometer (Forestry Suppliers, Jackson, MS, USA) during full leaf-out at the four cardinal directions around each pond. We categorized within-pond habitat variables into ten different groupings (e.g. emergent vegetation, submergent vegetation, cattails, coarse woody debris, duckweed, water shield, lily pads), and recorded the presence/absence of each type for all ponds in summer 2012. Slope of the pond basin was quantified by taking depth measurements at 1- and 2-m distances from the shoreline at multiple locations, calculating slope, and averaging values for each pond.

**Table 1:** Summary of the species composition in ephemeral ponds, permanent ponds without fish, and permanent ponds with fish in the area sampled at Fort Leonard Wood, Missouri.

Species	Ephemeral(n = 93)	Proportion	Species	Permanent w/o Fish (n = 75)	Proportion	Species	Permanent w Fish n = 26)	Proportion
Hylid	65	0.699	*Rana clamitans*	72	0.960	*Rana catesbeiana*	25	0.962
*Ambystoma maculatum*	60	0.645	*Rana catesbeiana*	67	0.893	*Rana clamitans*	24	0.923
*Notophthalmus viridescens louisianensis*	58	0.624	*Notophthalmus viridescens louisianensis*	64	0.853	*Acris blanchardi*	18	0.692
*Pseudacris crucifer*	56	0.602	Hylid	60	0.800	*Notophthalmus viridescens louisianensis*	17	0.654
*Ambystoma annulatum*	49	0.527	*Rana sphenocephala*	58	0.773	*Rana sphenocephala*	14	0.538
*Ambystoma opacum*	43	0.462	*Acris blanchardi*	54	0.720	Hylid	13	0.500
*Rana sphenocephala*	41	0.441	*Ambystoma maculatum*	54	0.720	*Ambystoma maculatum*	10	0.385
*Hyla chrysoscelis/versicolor*	26	0.279	*Pseudacris crucifer*	47	0.627	*Bufo spp*	9	0.346
*Rana clamitans*	24	0.258	*Hyla chrysoscelis/versicolor*	41	0.547	*Hyla chrysoscelis/versicolor*	8	0.308
*Bufo spp*	24	0.258	*Ambystoma annulatum*	38	0.507	*Pseudacris crucifer*	8	0.308
*Acris blanchardi*	20	0.215	*Ambystoma opacum*	31	0.413	*Ambystoma opacum*	3	0.115
*Rana catesbeiana*	18	0.194	*Bufo spp*	18	0.240	*Ambystoma annulatum*	2	0.077
*Pseudacris maculata*	17	0.183	*Rana palustris*	9	0.120	*Pseudacris maculata*	0	0.000
*Rana palustris*	5	0.054	*Pseudacris maculata*	2	0.027	*Rana palustris*	0	0.000

Proportion equals the number of ponds of particular hydroperiod type at which a species was detected during our surveys divided by the total number of ponds of that particular hydroperiod type during 2012 and 2013 combined.

### Analyses

We calculated amphibian larval abundance (cumulative for all traps and dip net sweeps at each pond over three days for each survey period, February-March and May-June) within each year (six days, maximum of 120 dip net sweeps and 120 trap checks per pond). Totals from the two sampling methods were combined after we saw no effect of survey method on abundance in a previous study [20]. We determined amphibian species richness and diversity index using the Shannon-Wiener’s H index by combining the two sampling periods for one estimate per year. We converted the Shannon-Wiener index into an effective number of species, which is simply an exponential transformation, and refer to it hereafter as diversity (see details in [26]). To assess relationships of diversity, richness and density with pond area, we compared the fit of linear and quadratic models for each response in each year separately using AIC criteria. We tested for overdispersion in each model, and found that the richness and abundance data showed overdispersion. For these we used distributions that best fit our data and added pond as a random effect to account for unique location effects. For abundance, we used generalized linear mixed effects models with a Poisson distribution and pond identity as a random effect to correct for overdispersion. The mean sampling date of a pond within each survey period (February-March or May-June) was also included as a random effect to account for repeated surveys of ponds. Sampling effort (i.e. total number of traps/dipnets) was included as an offset parameter. Thus, our response from these models was relative abundance of amphibians scaled to sampling effort, hereafter amphibian density. Richness was modeled with a generalized linear model using a negative binomial distribution, and diversity using a generalized linear mixed effects model with a Gamma distribution. To determine the pond area where amphibian density, richness, and diversity peaked, we resampled our data with replacement, and calculated the mean and 95% confidence intervals from 1000 bootstrap iterations.

Based on the above analysis, we found all three responses peaked at intermediate pond sizes (see Results). However, a substantial number of intermediate sized ponds also had low richness, diversity and abundance values. We therefore explored the within-pond factors that would potentially differentiate ponds of intermediate size that contributed to the peaks in density, richness, and diversity versus those that were below the peak. The data from 2012 and 2013 were pooled, using year as an additional random effect within the mixed effects models. We identified ponds within the peaked distribution if they were greater than the 50^th^ percentile of the density, richness, or diversity curves (S1 Fig). We then fit generalized linear mixed effects models with a binomial distribution to compare the ponds within the peak of the distribution with those below it. Independent variables in these models were percent canopy, presence of fish, hydroperiod, number of habitats present in each pond, density of invertebrate predators, and diversity of invertebrate predators. Invertebrate predator density and diversity were calculated as described for amphibians. We selected these parameters as they have been previously shown to influence amphibians in our study system [20]. We also have previously performed a Pearson’s correlation analysis of all explanatory variables, and eliminated one of the two covariates where r > 0.65 [20].

To assess the generality of our fitted models describing the effect of area on density, richness, and diversity we used the predicted sum-of-squares (PRESS) statistic and calculated a PRESS *R*-squared statistic from this [27]. We assessed the ability of binomial mixed effects models to correctly classify a pond as contributing to the peak in density, richness, and diversity by calculating the area under the receiver operating curve (AUC) from 10-fold cross validation repeated 100 times. All predictive performance measures were only calculated on the fixed effects, therefore they represent marginal model assessments.

## Results

**Fig 3:**
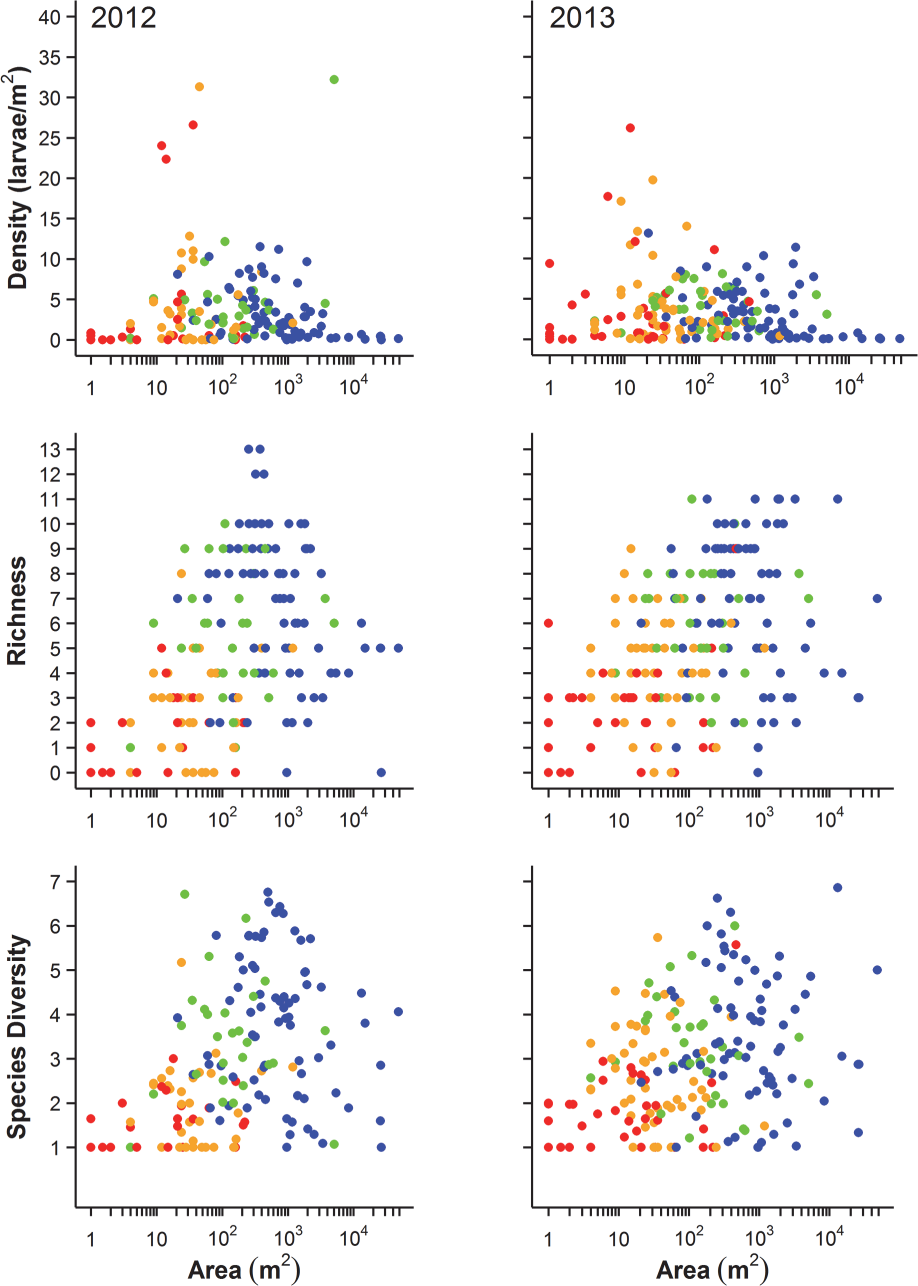
Species richness, effective number of species, and mean density of amphibians in ponds at Fort Leonard Wood, Missouri in relationship to pond area. Hydroperiod was assigned into four categories: ephemeral (red), summer (yellow), semi-permanent (green), and permanent (blue; see Methods). Each dot represents one pond.

Based on the entire data set, we captured a total of 14 species of pond-breeding amphibians in both years among all ponds (S1 Table). Individual ponds held as many as 13 species and reached larval densities of 35 per m^2^ (Fig 3). The average number of species per pond was 4.67 ± 0.25 for 2012 and 5.10 ± 0.21 for 2013 (Fig 3). Some ponds were unoccupied (13%- 2012, 6%- 2013) and other ponds had just 1–2 species (17%- 2012, 14%- 2013), primarily very small ponds and ruts (Fig 3). Species were predictably found in different pond types; Hylid frogs, comprised of *Hyla chrysoscelis/versicolor*, *Pseudacris crucifer* and *P*. *maculata*, and the salamanders *Ambystoma maculatum* and *Notophthalmus viridescens louisianensis* were detected at the highest proportion of ephemeral sites (> 62%), while *Rana catesbeiana* and *R*. *clamitans* were detected at > 92%, and *Acris blanchardi* were detected at > 69% of the permanent sites with fish (Table 1).

**Fig 4:**
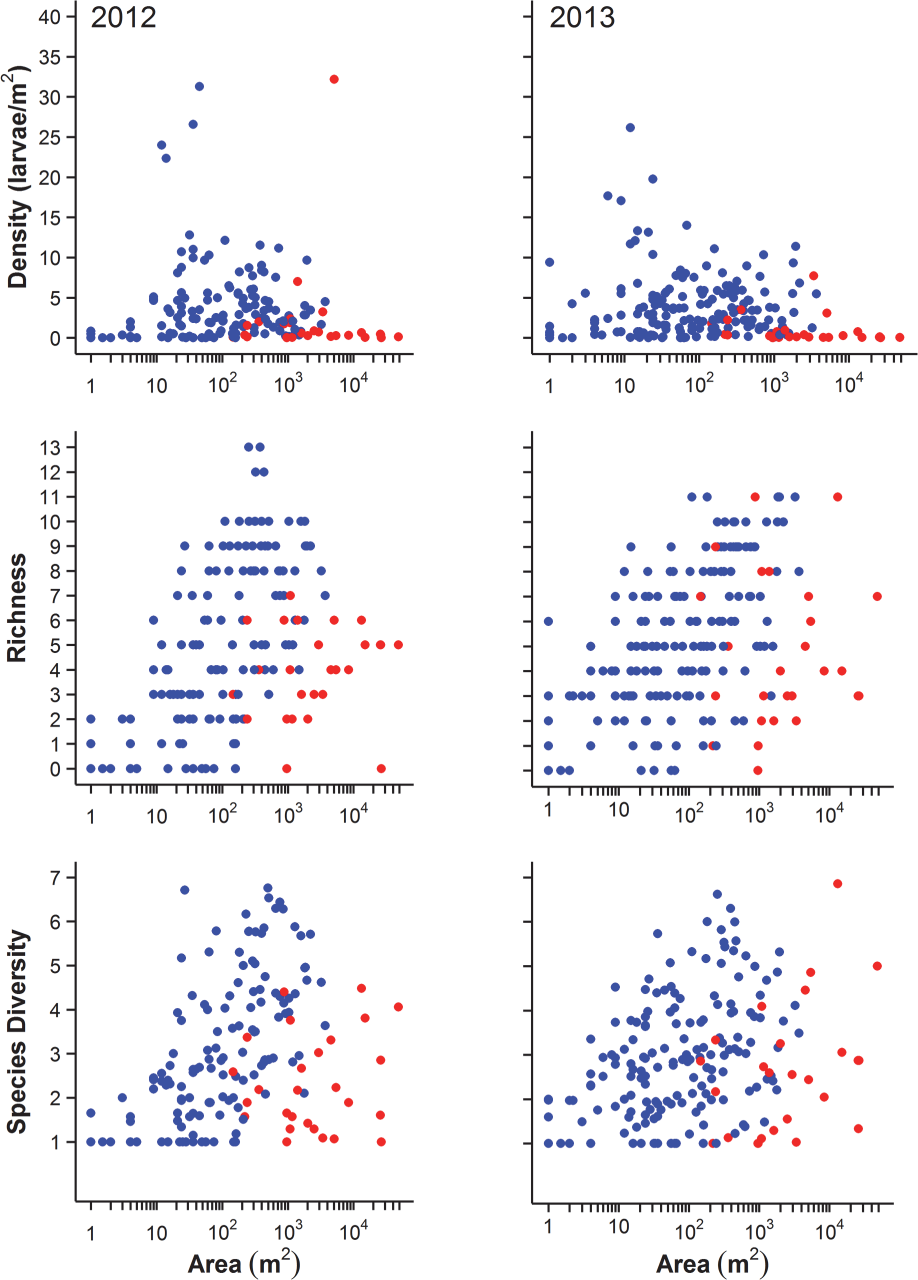
Species richness, effective number of species, and mean density of amphibians in ponds at Fort Leonard Wood, Missouri in relationship to pond area. Species richness, effective number of species, and mean density of amphibians across pond area in ponds at Fort Leonard Wood, Missouri in relationship to the presence (red) or absence (blue) of fish (see Methods). Each dot represents one pond.

Pond area varied significantly among hydroperiod categories (F_3,198_ = 5.93, P = 0.0007): mean size of ephemeral ponds was 42.8 m^2^, summer drying ponds was 76.5 m^2^, semi-permanent ponds was 437.5 m^2^, and permanent ponds was 3003.5 m^2^ (Fig 3). Ponds that had a permanent hydroperiod and contained fish were larger in area (7374.7 m^2^) compared to fishless ponds (334.7 m^2^; Fig 4). The largest ponds sampled contained primarily game fish but some contained up to five fish groups or genera (mosquitofish, *Gambusia* sp.; sunfish, *Lepomis* sp.; bass, *Micropterus* sp.; catfish, Siluriformes; and minnows, Cyprinidae).

**Fig 5:**
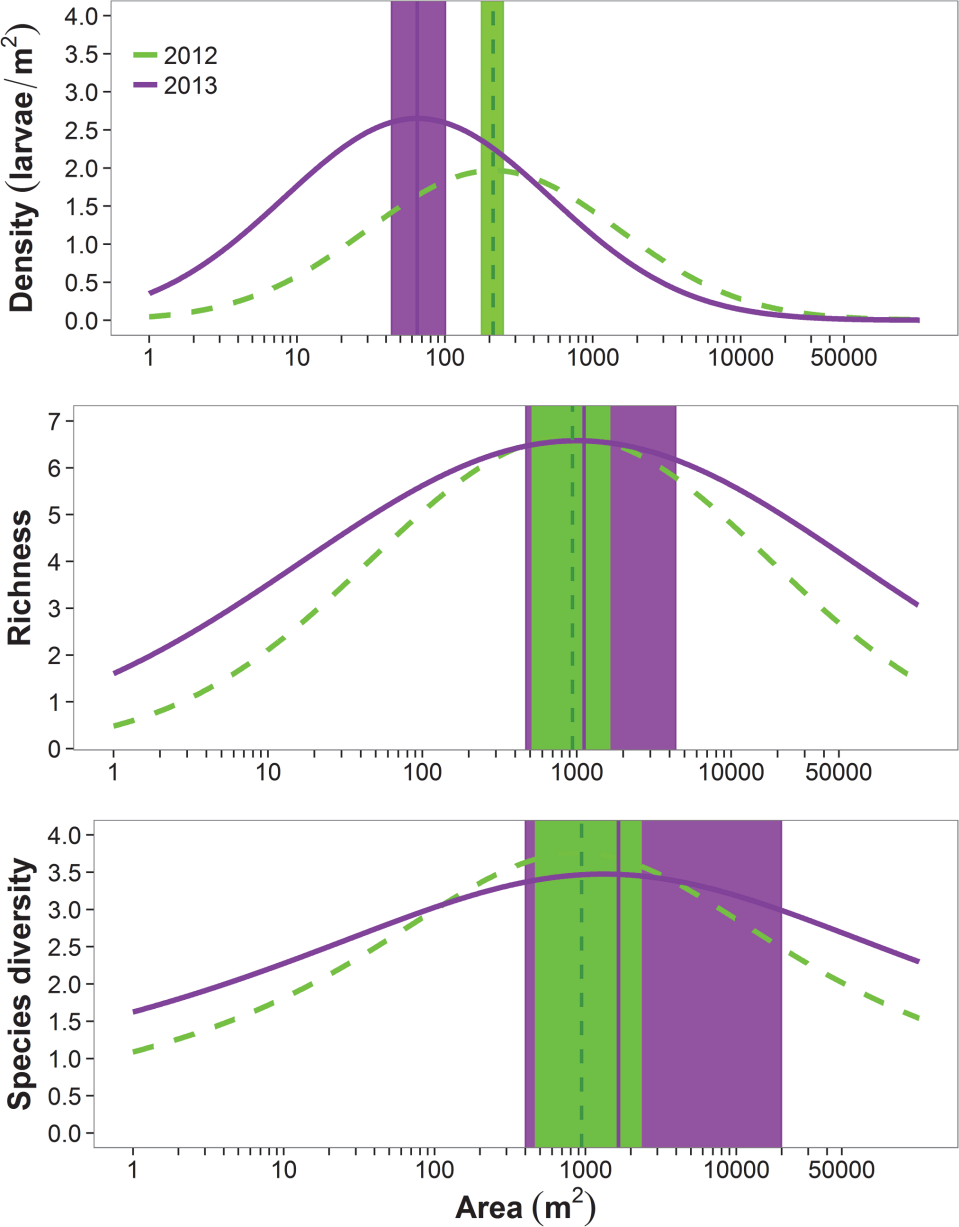
Model fit for species richness, effective number of species, and mean density of amphibians in ponds at Fort Leonard Wood, Missouri for 2012 and 2013 in relationship to log (pond area m^2^). Vertical lines indicate the pond area where the curve peaks, and shaded areas indicate 95% prediction intervals around this mean.

Density, species richness, and diversity reached their highest values at intermediate pond areas and in all cases a quadratic model fit significantly better than a linear model (Table 2 and Fig 5). Pond area at which richness and diversity peaked did not differ between 2012 and 2013, although the confidence intervals were slightly broader in 2013 (Fig 5). Peak density occurred at smaller pond areas than richness and diversity, and was different between years (pond areas of 211 m^2^ (95% CI = 177–248 m^2^) and 65 m^2^ (43–100 m^2^) in 2012 and 2013, respectively; Table 2 and Fig 5). The density, richness, and diversity of species declined as pond size reached the largest values of area (Figs 3, 4 and 5). Mixed effects models fit for years 2012 and 2013 combined had moderate ability to predict density, richness, and abundance with PRESS *R* ^2^ of 0.223, 0.251, and 0.170, respectively.

**Table 2:** Pond area at peak of response curve, parameter estimates (± S.E.) and model fit based on likelihood ratio tests for the best supported models of amphibian richness, diversity and abundance.

			Parameter estimates		
Year	Response	Pond Area at Peak Value (95%CI)	Intercept ± S.E.	Area ± S.E.	Area^2^ ± S.E.	Chi square	P value
2012	Richness	934 m^2^ (528 m^2^-1669m^2^)	-0.70±0.28	0.76±0.1	-0.056±0.01	40.04	<0.0001
	Diversity	1450 (578 m^2^-7942 m^2^)	0.50±0.39	0.82±16	-0.056±0.015	12.95	0.0003
	Density	211 m^2^ (177 m^2^-248 m^2^)	-2.95±0.59	1.36±0.23	-0.13±0.02	29.17	<0.0001
2013	Richness	1012 m^2^ (497 m^2^-6502 m^2^)	0.47±0.17	0.41±0.065	-0.03±0.006	22.96	<0.0001
	Diversity	3229 m^2^ (290 m^2^-17854 m^2^)	1.40±0.32	0.5±0.14	-0.031±0.014	5.28	0.02
	Density	65m^2^ (43 m^2^-100 m^2^)	-1.14±0.42	1.13±0.17	-0.091±0.017	28.79	<0.0001

In the next level of analysis, we used the upper 50^th^ percentile of the data in the density, richness, and diversity curves that represents the peak distribution to address why some ponds of intermediate area seemed to vary greatly in the response variables (Figs 3, 4 and S1 Fig). Using this subset of data also more directly compares ponds of similar area (eliminating 25% of the smallest and 25% of the largest ponds). We found that the presence of fish, invertebrate density, and predator diversity were significant drivers for all three responses within the middle range of pond areas (Table 3 and S2, S3, S4, and S5 Figs). Additionally, the amount of canopy was important only for amphibian density and the total number of aquatic habitats was important only for diversity of species (Table 3 and S2, S3, S4, and S5 Figs). Each of the binomial models was able to discriminate ponds contributing the peak. The mean AUC of the density model was 0.897 (0.882–0.911 CI), the mean AUC of the richness model was 0.860 (0.807–0.912 CI), and the mean AUC of the diversity model was 0.874 (0.823–0.925 CI). Although hydroperiod was not a significant predictor in the binomial mixed effects models (Table 3 and S2, S3, and S4 Figs), partially because this analysis eliminated the extreme hydroperiods, ponds contributing to the peak in density, richness, and diversity all had longer hydroperiods than ponds of similar size that were below the peak (Table 3 and S5 Fig).

**Table 3:** Summary tables of generalized linear mixed effects models fit with a binomial distribution.

**Density peak**	**Parameter**	**Estimate**	**S.E.**	**z-value**	***P***
	Intercept	2.022	0.245	8.264	<0.001
	**Canopy**	**0.529**	**0.148**	**3.575**	**<0.001**
	**Fish present**	**-2.909**	**0.543**	**-5.360**	**<0.001**
	Hydroperiod	-0.028	0.228	-0.124	0.901
	*Hydroperiod* ^*2*^	*-0*.*314*	*0*.*174*	*-1*.*808*	*0*.*071*
	*Number of habitats*	*0*.*300*	*0*.*168*	*1*.*783*	*0*.*075*
	**Predator density**	**0.453**	**0.165**	**2.746**	**0.006**
	**Predator density** ^**2**^	**-0.093**	**0.037**	**-2.541**	**0.011**
	**Predator diversity**	**0.441**	**0.126**	**3.501**	**<0.001**
	Predator diversity^2^	-0.062	0.091	-0.681	0.496
**Richness peak**	**Parameter**	**Estimate**	**S.E.**	**z-value**	***P***
	Intercept	1.472	0.370	3.977	<0.001
	Canopy	0.157	0.185	0.846	0.397
	**Fish present**	**-1.657**	**0.528**	**-3.141**	**0.002**
	Hydroperiod	0.402	0.348	1.157	0.247
	Hydroperiod^2^	-0.126	0.199	-0.635	0.526
	Number of habitats	0.271	0.190	1.423	0.155
	**Predator density**	**1.067**	**0.381**	**2.801**	**0.005**
	**Predator density** ^**2**^	**-0.197**	**0.107**	**-1.848**	**0.065**
	**Predator diversity**	**0.640**	**0.223**	**2.878**	**0.004**
	Predator diversity^2^	0.121	0.187	0.650	0.516
**Diversity peak**	**Parameter**	**Estimate**	**S.E.**	**z-value**	***P***
	Intercept	1.386	0.354	3.922	<0.001
	Canopy	0.238	0.219	1.085	0.278
	Fish present	-1.660	0.573	-2.897	0.004
	Hydroperiod	-0.114	0.476	-0.240	0.811
	Hydroperiod^2^	-0.361	0.246	-1.468	0.142
	**Number of habitats**	**0.746**	**0.221**	**3.373**	**0.001**
	**Predator density**	**1.363**	**0.396**	**3.440**	**0.001**
	**Predator density** ^**2**^	**-0.453**	**0.197**	**-2.299**	**0.021**
	**Predator diversity**	**0.480**	**0.236**	**2.036**	**0.042**
	Predator diversity^2^	0.233	0.199	1.175	0.240

Each model predicts whether a pond is contributing to the peaked distribution observed in density, species richness, or species diversity (Appendix B). All parameters are scaled and centered, so parameter estimates correspond to the relative effects of each parameter. Parameter estimates are on the logit scale. Bolded parameters are significant, while those in italics are near significant (α = 0.05).

## Discussion

Our results show a consistent and predictable pattern of peak larval density, richness, and species diversity of amphibians at intermediate pond area that partially supports the hypothesis of a balance between pond drying and predation. A similar pattern has now been demonstrated across several regions of the U.S. [28–30], including Missouri, and indicates that mechanisms structuring these pond communities are likely general and predictable [18]. This mechanistic understanding is a powerful tool for managing and protecting amphibian biodiversity as abiotic factors of breeding ponds can be coupled with biotic characteristics of species to understand where fitness is maximized [18]. We add to this understanding by including a robust assessment of abundance that is closely related to species success and the sustainability of populations.

Mechanistically, we found that small ponds or other water bodies have a short hydroperiod and a high probability of drying before most species complete egg or larval development. This disturbance due to pond drying excludes all but a few species with life history traits such as fast hatching eggs and short larval periods. Hylid frogs that include *Hyla versicolor/chrysoscelis*, *Pseudacris crucifer* and *P*. *maculata* are consistently found in these ephemeral ponds because their rapid development allows them to metamorphose before ponds dry and annual drying eliminates most predators. These species also avoid laying eggs or have poor larval survival in ponds that do not dry each year due to invertebrate predation [37,40,41]. Large, more permanent water bodies inflict a different disturbance in the form of fish that consume eggs and larvae before species complete development. Again, only species with anti-predator mechanisms that allow them to resist predation are successful in larger, permanent ponds. We consistently found *Rana catesbeiana*, *R*. *clamitans*, and *Acris blanchardi* in permanent ponds with fish because each of these species has anti-predator mechanisms such as distasteful larvae, avoidance behavior, or rapid growth to escape gape-limited fish predators [23,33]. Consistent species-specific responses such as these suggests that species’ autecological traits likely interact with the hydroperiod gradient [7,11]. Together, mechanisms of rapid drying and fish predation act to reduce density, richness, and diversity at either end of the pond area gradient and allow species maximum potential to successfully produce larvae at intermediate pond sizes.

Yet, we found that not all ponds that had intermediate areas were productive. In fact, many had low density, richness, and diversity (Figs 3 and 4). Results from ponds represented in the peak show that fish, invertebrate density and diversity, number of available aquatic habitats, and canopy are additional factors that drive density, richness, and diversity of ponds up or down, independent of area. While we acknowledge that our analysis does not disentangle all the ecological drivers of diversity or establish cause and effect, such as species interactions, we do provide a clear pattern of high diversity at intermediate pond sizes as previously predicted [12,18,31]. Ponds in the Midwestern U.S. have physical characteristics primarily related to area, depth, volume, substrate, and drainage that allow them to fill and retain water for varying lengths of time. The hydroperiod gradient created by these physical characteristics is a strong predictor of the species of predators and prey present in freshwater systems, including amphibian communities [18,32]. However, hydroperiod is correlated with pond area, and more recently pond area has been shown to be a predictor of amphibian richness, especially at broader spatial scales [29,30]. We also found that pond area was a strong predictor of diversity and density among ponds across our landscape. This indicates that pond area had a moderate ability to serve as a simple, single predictor of density, richness, and diversity. This is important because pond area can be measured accurately and easily obtained from remote sensing imagery over a large landscape compared to labor intensive hydroperiod measurements. However, we found that ponds with the greatest density, richness, and diversity had longer hydroperiods than similar sized ponds with low density, richness, and diversity. Conflicting results across studies that have used either pond area or hydroperiod are likely due to the fact that pond area is a coarser environmental gradient in which the filling and drying processes take place to determine hydroperiod. Further, our study intentionally included waterbodies of all sizes and hydroperiods to provide data along the broadest environmental gradient possible. Despite its ease of measurement and advantage in managing large landscapes, pond area alone cannot account for differences in depth, permeability of soils, or watershed size that additionally affect hydroperiod length. Pond area is really a surrogate for all aspects of disturbance such as intensity, timing, duration, extent, and frequency that can affect species responses [10]. A particular species may respond more strongly to one aspect of disturbance than another that is not accounted for by pond area alone. Our sampling intentionally included ditches and road ruts that can hold water for longer periods of time than predicted based on area alone and were found to be more productive for amphibians than expected. If such highly artificial waterbodies are excluded from sampling based on their small size or an *a priori* expectation that they are not productive for amphibians, then the full gradient of breeding resources could be biased and species missed. We fully acknowledge that data on the actual number of days a pond is full, date of filling and drying, or interval between drying may provide a finer environmental gradient in which to measure species responses. This finer resolution of data might explain why some intermediate sized ponds had very low diversity and abundance, and show how species respond to different aspects of disturbance. We found that ponds of intermediate areas with longer hydroperiods that lacked fish may facilitate a build up of invertebrate predators if drying did not occur each year (i.e. long interval between drying) and reduce amphibian diversity and density [33]. Further, it is very likely that well-established nonconsumptive behaviorally-mediated avoidance effects played a role in driving density, richness, and diversity down in such larger ponds [15,34–37]. Yet, similarly sized ponds that dry annually may have fewer invertebrates and are more likely to have a high density, richness, and diversity of amphibians at intermediate levels of invertebrate predator density (S2, S3, and S4 Figs).

We assumed that pond drying and colonization by fish represented two different disturbance processes causing declines in density, richness, and diversity of amphibians. However, they are not often discussed as separate processes, yet it is empirically [12,15,38–42] and conceptually well-founded [2,5]. Both disturbance processes are driven by natural stochastic weather events. Natural colonization of ponds by fish is indirectly related to hydroperiod through high rainfall and flooding events that cause hydrological connections to perennial streams or waterbodies facilitating overland movement of fish into ponds. Human stocking of fish into ponds with more permanent hydroperiods can also yield the same negative ecological effects on amphibians [43–46]. Natural drying resulting from drought, low rainfall, high temperature, and/or high levels of evapotranspiration. In large ponds, it eliminates fish and other invertebrate predators introduced by a flood event or their buildup after a series of wet years. In larger, more permanent ponds, drying takes longer, and fish are retained for longer periods of time after colonization, often at intervals of decades which effectively excludes all but one or two species of amphibians (such as bullfrogs) that can coexist and are even facilitated by fish [44]. In small ponds, however, if drying occurs too fast, only a few species with rapid development and short larval periods, such as spadefoot toads, will metamorphose early and persist [14]. Thus, in most cases the species occupying ponds are directly related to the intensity, frequency, and duration of weather events. For example, regional droughts lasting a number of years, can cause reproductive failure long enough to prevent recruitment and result in local extinction of species at some ponds, especially for short-lived species [16]. Perturbations due to land use or climate change, for example, can increase the rate of drying, increase drying duration or frequency, and shorten hydroperiods, excluding more species. Changes in climate may also affect the timing or date of onset of drying that can interact with species life histories (e.g. breeding phenology) to negatively affect some species while allowing others to persist [24]. However, once inundated following drying, these ponds can be rapidly colonized by some anuran species normally excluded by fish and yield large numbers of juveniles (e.g. chorus frogs [47]). The interaction each year between stochastic weather factors and pond area yield different outcomes both within individual ponds (temporal effect) and among ponds (spatial effect) on the landscape. Understanding this balance between drying and flooding due to current weather or changing climate conditions becomes critical for developing effective management strategies for amphibians.

Our results may partially fit within multiple diversity models [2–7], however, we suggest that the interaction among disturbance, productivity, consumption, and species’ traits is likely necessary to predict patterns of amphibian diversity in ponds rather than any single gradient. Seasonal ponds have been hypothesized to be valuable reproductive resources for amphibians responsible for maintenance of the aquatic larval stage in complex life cycle species because of the flush of nutrients, and high primary and secondary productivity in ponds [13]. The smallest ponds are only suitable for a small number of species because as a “reproductive resource” these ponds have a short duration and can only support those species with a short larval period. As ponds get larger, they hold water longer and allow more successful reproduction and more individuals of each species to be present. Studies that have examined pond gradients have found both the number of species and number of individuals increased with pond size or pond duration (hydroperiod) in a linear fashion [16,30,49,50]. Werner et al. [30] indicated that as the number of individuals and number of species increase in ponds there was little signal from biotic factors such as competitive exclusion being important relative to abiotic factors, and they suggested that saturation of pond habitat seldom occurs which is what may allow reproductive productivity to increase with pond size. We also show that a greater number of habitats within a pond increases the diversity of amphibians within intermediate sized ponds, or at least, increases the probability that a pond is in the peak of the distribution (S2, S3, S4, and S5 Figs). Large ponds may be more heterogeneous, providing deep and shallow areas, with and without vegetation to accommodate species-specific needs for oviposition sites, thermoregulation, or predator avoidance. They may also provide more space and food resources for more species and individuals (especially larger predatory salamander larvae), although this is untested. Thus, larger ponds may be considered better for the production of larvae both in terms of number and diversity. However, at the extreme end of the size gradient, consumption via predation, in our system, is likely the driving mechanism where the largest pond sizes (long hydroperiods or long intervals between drying) allow a buildup of invertebrate and fish predators, which can occur rapidly [17,33,51]. Further, we found that the biotic factor invertebrate predator density may play a role in the richness and diversity of the amphibian community, with these responses reaching their peak at intermediate values (S2, S3, S4, and S5 Figs). Few studies have explicitly included these largest and most permanent ponds to document the effects of consumption by predators, so studies may show no decline at this extreme end, thereby assuming that diversity-area relationships only increase linearly. Our results that include ponds up to 42,000 m^2^ and other studies clearly show that fish reduce the number of species and individuals [30,49]. Additionally, previously discussed behaviorally-mediated avoidance effects of both invertebrate and fish predators by amphibians can have a significant nonconsumptive effect in larger ponds with longer hydroperiods that was not included in the Worms et al. [48] model. Thus, multiple factors likely account for the unimodal peak in density, richness, and diversity we show at intermediate size ponds.

Our results have several important implications for conservation and management of amphibians. We suggest that mitigation and restoration efforts for amphibians focus on intermediate-sized ponds ranging from 200 to 4000 m^2^ to maximize abundance, richness and diversity. Ponds in this size range appear better able to recruit large numbers of juveniles into adult populations and may represent source ponds for dispersers that are important for rescuing sink ponds and sustaining regional metapopulations [52,53]. However, inclusion of the smallest and most ephemeral ponds is still an important management recommendation to protect the full range of amphibian diversity, including species exclusively adapted to highly ephemeral breeding sites, and to maintain a high density of ponds to maximize connectivity [28,54]. Similar recommendations will also be important for including other taxa such as invertebrates that depend on ephemeral environments. Further, because annual weather patterns that affect rainfall and temperature can strongly interact with pond attributes to determine hydroperiod [17] or other disturbance factors, conservation and management efforts should recognize the value of restoring or mitigating a full gradient of pond sizes and hydroperiods, especially larger, fishless ponds, to ameliorate any effects due to climate change and increased intensity or duration of droughts [24,55]. We hope our results increase understanding of how different aspects related to pond size affect amphibian diversity and persistence of species, and provide a framework based on pond area for developing effective restoration and conservation solutions at larger spatial scales. However, we emphasize that area alone does not provide complete insight concerning restoration and conservation for individual ponds or particular species of concern, thereby suggesting that hydroperiod, filling and drying time, and drying interval must also be measured in particular cases to truly maximize abundance, richness, and diversity of amphibians.

## Supporting Information

S1 Fig Ponds included in peak analysis. Response curves of mixed effects models using larval density, species richness, and species diversity as response variables highlighting ponds above and below peak values. The curves drawn are fit to both the 2012 and 2013 data combined. The models utilized are the same as described in the text, except that year is included as an additional random effect. Ponds were considered to be contributing to the peaked response in each curve if they were greater than the 50^th^ percentile of the distribution (green boxes). The purple box in each figure encompasses the region below the peak. Values inside each box indicate the number of observations.
(TIF)

S2 Fig Response curves for each parameter contributing to the peak based on significant parameters from the fitted models—density. While the percent canopy over the pond, number of invertebrate predator species, and invertebrate predator density all have significant effects in the density model, it can be seen that each contributes minimally to the probability of a pond being included in the peak. This in part may be due to the fact that abundance (density) can be difficult to accurately quantify in the field, but may also indicate that when amphibians select a pond to breed in, equal reproductive effort is allocated to each. The probability of being in the peak is greatest at a predator density of 10.66 m^-2^.
(TIF)

S3 Fig Response curves for each parameter contributing to the peak based on significant parameters from the fitted models—richness. Ponds with intermediate invertebrate predator densities and greater invertebrate predator diversity contribute to the peak in amphibian richness. The probability of being in the peak is greatest at a predator density of 12.23 m^-2^.
(TIF)

S4 Fig Response curves for each parameter contributing to the peak based on significant parameters from the fitted models—diversity. Ponds with a greater number of habitat types, higher density of invertebrate predators, and greater predator diversity had the greatest probability of contributing the peak in amphibian diversity.
(TIF)

S5 Fig Hydroperiod of ponds in the peak. Plots showing mean hydroperiod with 95% confidence intervals for ponds contributing to the peak (green box, S1 Fig) or below the peak (purple box, S1 Fig). Ponds contributing to the peak in each response had, on average, a longer hydroperiod than similar sized ponds that were not contributing to the peak. Comparisons between groups were made using mixed effects models with hydroperiod as the response, and date (for density model) or year (richness and diversity models) as a random effect. Reported t-values in the figures are estimated from the fixed effects model.
(TIF)

S1 Table Amphibians and invertebrates sampled. All amphibians (Caudata, Anura) and invertebrate predators with their common names that were captured during larval aquatic sampling of ponds at Fort Leonard Wood, Pulaski County, Missouri.
(DOCX)
