# Supplementary figures and images for: Intermediate Pond Sizes Contain the Highest Density, Richness, and Diversity of Pond-Breeding Amphibians
Source: PLoS One. 2015 Apr 23;10(4):e0123055. doi: 10.1371/journal.pone.0123055 (PMC4408075; doi:10.1371/journal.pone.0123055)

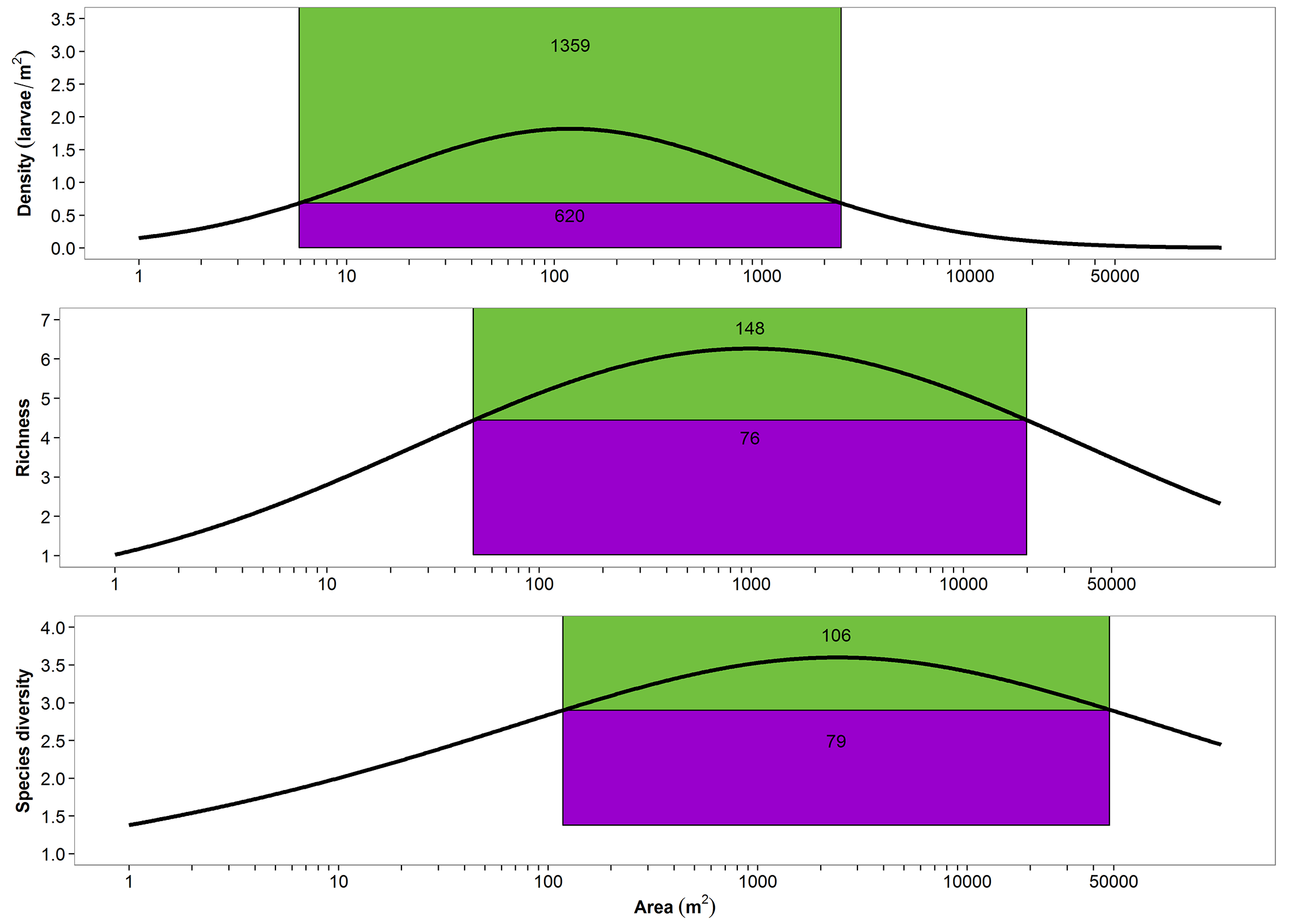

Supplement: S1 Fig — Response curves of mixed effects models using larval density, species richness, and species diversity as response variables highlighting ponds above and below peak values. The curves drawn are fit to both the 2012 and 2013 data combined. The models utilized are the same as described in the text, except that year is included as an additional random effect. Ponds were considered to be contributing to the peaked response in each curve if they were greater than the 50th percentile of the distribution (green boxes). The purple box in each figure encompasses the region below the peak. Values inside each box indicate the number of observations. (TIF) [file pone.0123055.s001.tif]

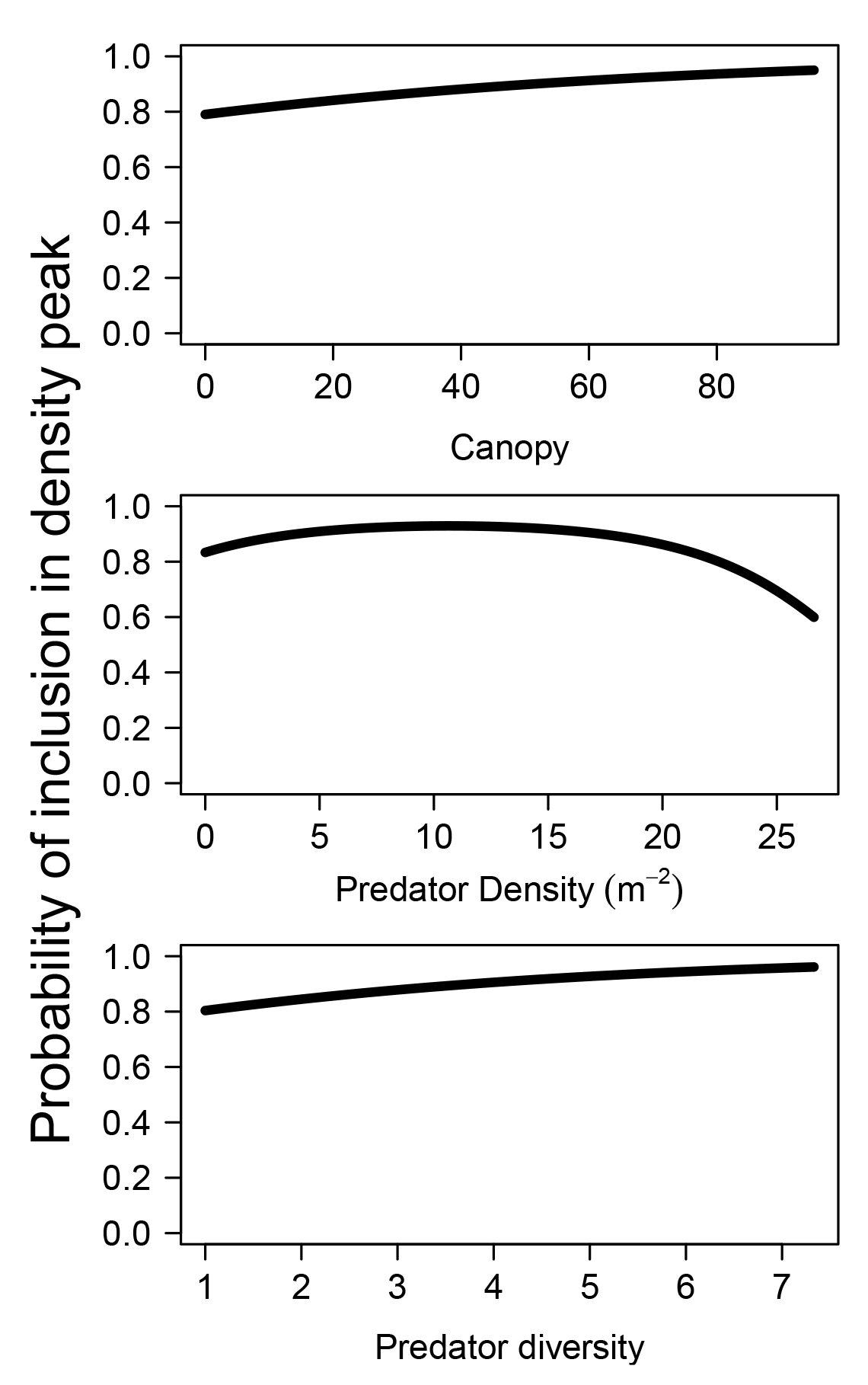

Supplement: S2 Fig — While the percent canopy over the pond, number of invertebrate predator species, and invertebrate predator density all have significant effects in the density model, it can be seen that each contributes minimally to the probability of a pond being included in the peak. This in part may be due to the fact that abundance (density) can be difficult to accurately quantify in the field, but may also indicate that when amphibians select a pond to breed in, equal reproductive effort is allocated to each. The probability of being in the peak is greatest at a predator density of 10.66 m-2. (TIF) [file pone.0123055.s002.tif]

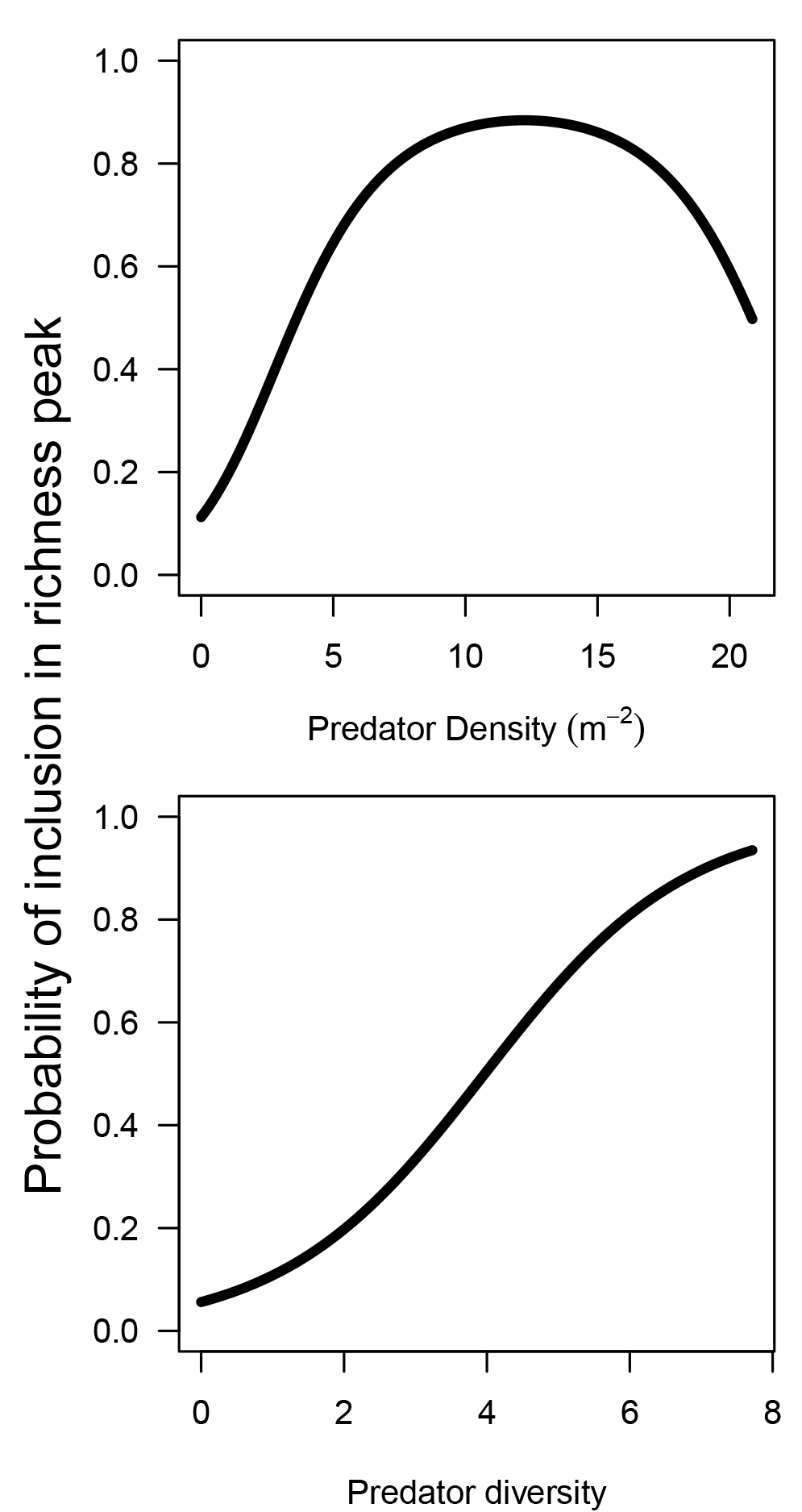

Supplement: S3 Fig — Ponds with intermediate invertebrate predator densities and greater invertebrate predator diversity contribute to the peak in amphibian richness. The probability of being in the peak is greatest at a predator density of 12.23 m-2. (TIF) [file pone.0123055.s003.tif]

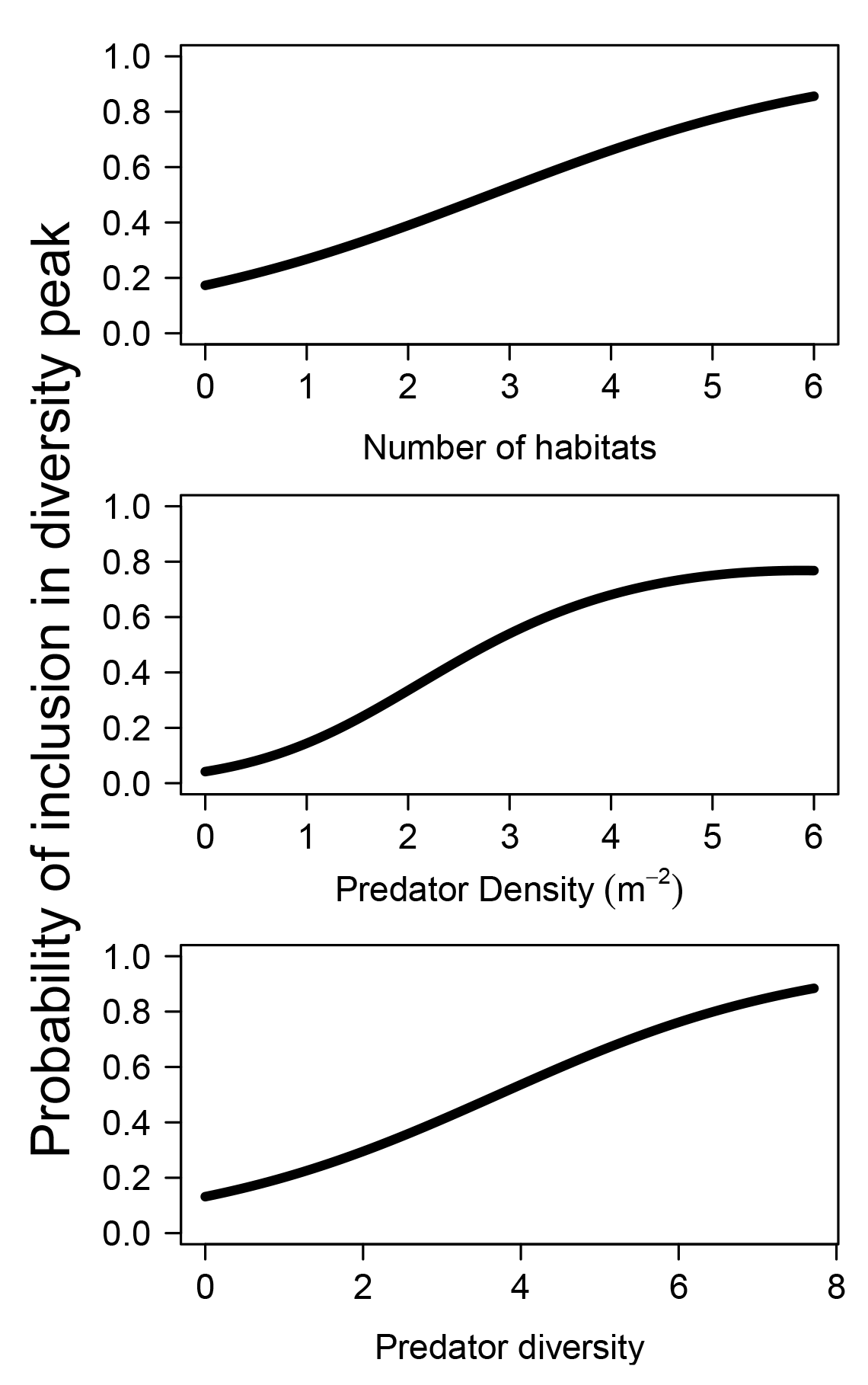

Supplement: S4 Fig — Ponds with a greater number of habitat types, higher density of invertebrate predators, and greater predator diversity had the greatest probability of contributing the peak in amphibian diversity. (TIF) [file pone.0123055.s004.tif]

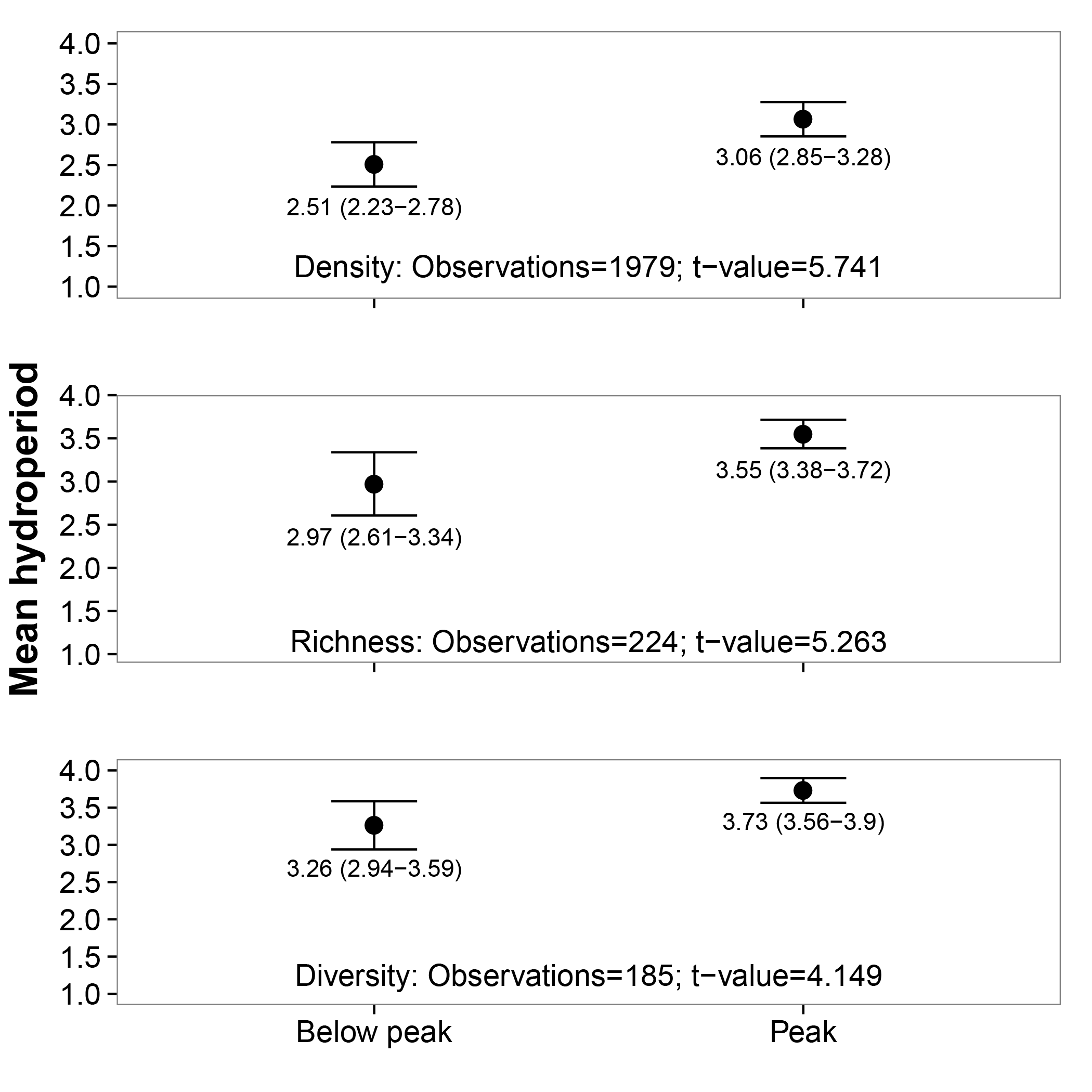

Supplement: S5 Fig — Plots showing mean hydroperiod with 95% confidence intervals for ponds contributing to the peak (green box, S1 Fig) or below the peak (purple box, S1 Fig). Ponds contributing to the peak in each response had, on average, a longer hydroperiod than similar sized ponds that were not contributing to the peak. Comparisons between groups were made using mixed effects models with hydroperiod as the response, and date (for density model) or year (richness and diversity models) as a random effect. Reported t-values in the figures are estimated from the fixed effects model. (TIF) [file pone.0123055.s005.tif]
