# Supplementary material for: Intermediate Pond Sizes Contain the Highest Density, Richness, and Diversity of Pond-Breeding Amphibians
Source: PLoS One. 2015 Apr 23;10(4):e0123055. doi: 10.1371/journal.pone.0123055 (PMC4408075; doi:10.1371/journal.pone.0123055)
Supplement: S1 Table — All amphibians (Caudata, Anura) and invertebrate predators with their common names that were captured during larval aquatic sampling of ponds at Fort Leonard Wood, Pulaski County, Missouri. (DOCX) [file pone.0123055.s006.docx]

Scientific name Common name

________________________________________________________________________

| Caudata: |  |
| --- | --- |
| *Ambystoma annulatum* | Ringed Salamander |
| *Ambystoma maculatum* | Spotted Salamander |
| *Ambystoma opacum* | Marbled Salamander |
| *Notophthalmus viridescens louisianensis* | Central Newt |
| Anura: |  |
| *Acris blanchardi* | Blanchard's Cricket Frog |
| *Bufo (Anaxyrus) americanus* | American Toad |
| *Bufo (Anaxyrus)fowleri* | Fowler's Toad |
| *Hyla chrysoscelis* | Cope's Gray Treefrog |
| *Hyla versicolor* | Gray Treefrog |
| *Pseudacris crucifer* | Spring Peeper |
| *Pseudacris maculata* | Chorus Frog |
| *Rana catesbeiana (Lithobates catesbeianus)* | Bullfrog |
| *Rana clamitans (Lithobates clamitans)* | Green Frog |
| *Rana palustris (Lithobates palustris)* | Pickerel Frog |
| *Rana sphenocephala (Lithobates sphenocephalus)* | Southern Leopard Frog |

Inverbrate predator classifications:

Aeshnidae darner dragonflies

Anisoptera non-aeshnid dragonflies

Belostomatidae giant water bugs

*Carydalus* spp. hellgrammites

Corixidae water boatmen

Dystiscidae predaceous diving beetles

Hydrophlidae water scavenging bugs

Hirudinae leeches

*Oronectes puncitmanus* spot-handed crayfish

Naucoridae creeping water bugs

Nepidae water scorpions

Notonectidae backswimmers

Tabanidae horseflies

Zygoptera damselflies

______________________________________________________________________
